# Case Series of COVID-19 Asymptomatic Newborns With Possible Intrapartum Transmission of SARS-CoV-2

**DOI:** 10.3389/fped.2020.568979

**Published:** 2020-09-29

**Authors:** Jean-Michel Hascoët, Jean-Marc Jellimann, Cedric Hartard, Apolline Wittwer, Hélène Jeulin, Patricia Franck, Olivier Morel

**Affiliations:** ^1^Division of Neonatology, Maternite Regionale, CHRU Nancy, EA 3450 Lorraine University, Nancy, France; ^2^Virology Unit, Department of Microbiology, Nancy, France; ^3^Lorraine University, CNRS, LCPME, Nancy, France; ^4^Laboratory of Biology, CHRU Nancy, Nancy, France; ^5^Department of Gynecology and Obstetrics, Maternite Regionale CHRU Nancy, Nancy, France

**Keywords:** newborn, COVID-19, vertical transmission, antibodies, case report, pregnancy

## Abstract

**Background:** Despite the pandemic, data are limited regarding COVID-19 infection in pregnant women and newborns. This report aimed to bring new information about presentation that could modify precautionary measures for infants born of mothers with a remote history of COVID-19.

**Methods:** We report two infants with possible maternofetal transmission, and four mothers without immunologic reactions. Data were collected from the patient files.

**Results:** One mother exhibited infection signs 10 days before uncomplicated delivery, with negative RT-PCR and no antibody detection thereafter. Another mother exhibited infection 6 weeks pre-delivery, confirmed by nasopharyngeal swab testing with positive RT-PCR, and positive antibody detection (IgM and IgG). Both newborns were asymptomatic but tested positive for nasopharyngeal and stool RT-PCR at 1 and 3 days of age for the first one and at 1 day of age for stool analysis for the second one. Two additional mothers exhibited infection confirmed by positive RT-PCR testing at 28- and 31-days pre-delivery but did not present detectable antibody reaction at the time of delivery.

**Conclusion:** These observations raise concerns regarding contamination risk by asymptomatic newborns and the efficacy of immunologic reactions in pregnant mothers, questioning the reliability of antibody testing during pregnancy.

## Introduction

Since early December 2019, we have witnessed the global spread of a novel coronavirus epidemic outbreak caused by SARS-CoV-2. As of June 28, 2020, over 9,580,000 patients have been infected, including about 163,000 confirmed cases in France. However, limited data are available regarding pregnant women with COVID-19 infection and their newborns (1). Notably, evidence of *in utero* vertical transmission is difficult to assess and remains controversial (2). Prior reports include one infant with elevated IgM at birth, suggesting antenatal infection (3), and one infant with a positive real time-polymerase chain reaction (RT-PCR) test and negative antibody reaction (4). These sparse findings may be related to limitations associated with the sensitivity of diagnostic tests, the delay between testing and the onset of symptoms (5–7), and/or unexpected clinical and immunological reactions to this novel viral infection (6).

The Grand-Est area of France has been heavily affected, and we have handled several cases in which infants have been born from SARS-CoV-2-infected mothers. Consistent with previously reported data (1–9), no case has involved newborn morbidity that might be related to the virus. Until now, we did not diagnose any infant with evidence of intrauterine infection. Here, we report two cases involving possible maternofetal asymptomatic vertical transmission and no antibody detection in mothers.

## Cases Description

On March 22, a woman presented with a fever, moderate cough, and profound asthenia consistent with COVID-19 infection. Both the mother and her spouse were healthcare providers in charge of COVID-19-infected patients. RT-PCR analysis of a single nasopharyngeal swab yielded negative results which does not exclude COVID-19 infection (10). For this test, viral RNA was extracted using the NucliSens® solution (bioMerieux France), and amplified via RT-PCR protocols developed by the French National Reference Center for Respiratory Viruses, Institut Pasteur, Paris, France. The patient's symptoms progressively resolved after 2 days.

On April 2nd, the mother underwent an uncomplicated labor and vaginal delivery, consistent with previous reports (11). Baby-boy T was born at 39 gestational weeks (Table 1). Fetal echography examinations had revealed transposition of the great vessels and interventricular communication. Immediately after birth, a Rashkind atrioseptostomy through the umbilical vein was attempted and failed. Since the baby had an overall good clinical condition and a SaO_2_ of between 80 and 89% while spontaneously breathing room air, it was decided not to make another attempt. Alprostadil perfusion at 0.02 mcg/kg/min was initiated, and the baby was prepared for transfer to surgery. Although the infant exhibited no signs suggesting viral infection, nasopharyngeal swab testing was performed 10 h after birth because the surgeons had made this procedure mandatory for all infants before surgery. The SARS-CoV-2 RT-PCR analysis was positive, and the baby was placed in complete isolation in a negative pressure room.

**Table 1:** Infants' Characteristics.

	**Baby boy T**	**Baby girl D**
Birth weight	2,740 g	3,170 g
APGAR score (1/5 min)	8/8	9/10
Clinical history	Transposition of the great vessels	Antenatal cardiac disproportion
RT-PCR	Positive in pharynx at 10 h	Positive in stools on day 1
	Positive in stools on day 3	Negative in pharynx on day 3
Red blood cell count	4.8 T/L	6.9 T/L
Reticulocytes	182 G/L	272 G/L
White blood cells	18.58 G/L	9.37 G/L
Neutrophils	10.57 G/L	6.31 G/L
Lymphocytes	4.72 G/L	1.62 G/L
Monocytes	2.21 G/L	1.21 G/L
Eosinophils	0.20 G/L	0.11 G/L
Basophils	0.17 G/L	0.06 G/L

On April 5, new nasopharyngeal testing was performed for both the mother and the baby, and both were negative for SARS-CoV-2 RNA. On the same day, RT-PCR analysis was performed using samples of breast milk and stool (from both the mother and newborn), yielding positive results for the baby's stool, negative results for breast milk, and inconclusive results for the mother's stool due to the presence of inhibitors. The baby was initially fed with expressed milk from the mother, and was then directly breastfed after the mother was trained in our specific procedure. This procedure included wearing a surgical mask before and throughout breastfeeding, as well as thoroughly washing her hands, using hydro-alcoholic gel after her mask manipulation, and cleaning her breast immediately before and washing her hands again before and after having breastfed the baby. A first serology evaluation on April 5th, 15 days after the first symptoms, (Biosynex Covid-19 BSS) was negative for IgM and IgG both in mother and infant, and negative for IgA and IgG (SARS-CoV-2 IgA/IgG, EUROIMMUN Elisa method) in the mother. On April 6th, Baby-boy T was transferred to surgery, and underwent an arterial switch operation. Evolution was good and the infant returned for follow-up on April 10th. Nasopharyngeal analysis was repeated and the results were negative for both the mother and the baby, who was discharged on April 12th. The infant had an outpatient follow-up visit on April 22nd at 3 weeks of age. Infant nasopharyngeal analysis was negative, as was a second serology evaluation for both the mother and infant, 32 days after the first maternal symptoms. Physical examination was normal for the infant's age. Weight was 3,180 g, up 440 g from birth. The infant showed no signs of pulmonary or digestive disorder, and neurodevelopment was as expected at this postnatal age. Finally, baby-boy T had a last outpatient follow-up visit on May 20th at 7 weeks of age. Physical examination was strictly normal, RT-PCR control was negative and no antibodies were detected by the assays used in our laboratory.

The second mother had COVID-19 infection with positive RT-PCR results on March 17th. She exhibited fever and profound asthenia for 10 days. On April 27th, she underwent an uncomplicated labor and vaginal delivery, and was considered cleared of COVID-19 as it was 6 weeks after the onset of infection. Baby-girl D was born at a gestational age of 39 weeks (Table 1). Fetal examinations had revealed antenatal cardiac disproportion, but serial echocardiography up to ductus closure excluded an aortic coarctation.

The infant was placed in complete isolation, in a negative pressure room, from birth while awaiting the test results, as per our new procedure. Indeed, based on asymptomatic Baby-boy T, we instituted a policy of systematic COVID-19 testing for all newborns whose mothers had an history of COVID-19 infection. Thus, although the mother was considered cleared at 6 weeks after the onset of infection, which was confirmed by negative nasopharyngeal and stool SARS-CoV-2 RT-PCR tests after delivery, we tested stool and pharynx swab samples from asymptomatic baby-girl D. The baby was initially fed with expressed milk from her mother, and was then directly breastfed following the above-described procedure. RT-PCR testing of the breast milk yielded negative results for SARS-CoV-2. However, testing of the baby's stool yielded positive results on day 1. Testing of pharynx swabs yielded negative results on day 3. IgG was detected in the mother and newborn, and IgM in the mother only (Biosynex Covid-19 BSS). On June 10th, baby-girl D had an outpatient follow-up visit, 45 days from birth, and 12 weeks from maternal symptoms and positive PCR. Physical examination was normal for the infant's age with no sign of pulmonary or digestive disorder, and she demonstrated appropriate neurodevelopment. She was completely breastfed. PCR control was negative as well as IgM detection but IgG detection was still positive.

Two additional babies and their mothers were tested at birth because the mothers had symptomatic infection, documented with positive SARS-CoV-2 RT-PCR results, at 28 and 31 days before delivery, respectively. The babies did not present any clinical sign of infection, they were negative for RT-PCR testing, and both mothers were negative for antibody detection.

## Discussion

These reports raise more questions than they provide clear answers. Despite the first newborn was asymptomatic and the screening performed as part of routine systematic testing, SARS-CoV-2 RNA detection through early nasopharyngeal sampling and the persistent detection of virus in stool strongly suggest possible vertical maternofetal infection. The physician and nurse in charge of the infant were screened negative and had negative antibody detection 9 and 12 weeks after delivery respectively. There was no other newborn in the NICU with infection or suspected infection at that time, and the infant had no contact with any other possible source of contamination. Finally, although Baby-boy T's infection was confirmed by positive results in nasopharyngeal sampling and stool analysis, antibodies were not detected at 3 and 7 weeks from PCR positive results by the assays used in our laboratory. This is consistent with Alzamora's report of a newborn infant with positive RT-PCR results at 16 h after birth, and negative antibody reaction (4). It has been previously reported that a significant percentage of patients with positive RT-PCR results exhibit negative results on IgM antibody tests, possibly due to host factors that influence the antibody response to SARS-CoV-2 (12).

The second newborn was screened because her mother had a remote history of COVID-19 infection, despite she was considered cleared of the virus at 6 weeks after documented infection and not excreting the virus and the time of delivery. Again, the physician and nurse in charge of the infant were screened and not infected, and we had no other newborn in our NICU with infection or suspected infection at that time. Additionally, the early detection of virus in stool samples from both babies excludes horizontal contamination from other unknown source (12). Indeed, in a review about the possibility of fecal transmission, Tian et al. (13) reported that positive fecal results occur at least 2–5 days after positive sputum PCR. Tang et al. (14) described an asymptomatic child who exhibited viral positivity in stool at 17 days after virus exposure. Therefore, one may conclude that the case of Baby-boy T was highly suspected to involve vertical maternofetal infection, and that Baby-girl D was infected via possible vertical antepartum maternofetal transmission. Baby-girl D had positive IgG detection, probably through passive antenatal transfer. Of note, both mothers were not excreting the virus at the time of delivery. However, despite very unlikely a contamination can never totally be excluded.

These observations raise concerns regarding the risk that healthcare providers may be contaminated through pauci- or asymptomatic newborns that excrete the virus in respiratory secretions or stool (4). Baby-boy T presented with a moderate fever starting 18 h after birth and lasting 72 h; however, he received prostaglandins treatment for his cardiopathy at that time and this treatment was the most probable cause of fever (15). The risk of contamination through stools cannot be excluded, and the viral RNA may be present in stools for longer than in nasopharyngeal excretion (13, 16). Notably, Baby-girl D was negative in her pharynx tests. Thus, one should be aware of this risk and all infants—regardless of symptoms, and whether they are born from mothers with infection or suspected infection—should be tested in both pharynx and stool, and considered at risk for contamination. Isolation and all precautionary measures for infected neonates should be maintained until two negative tests performed 24 h apart (4, 17).

The first mother did not develop anti-SARS-CoV-2 IgM nor IgG, and assay for IgA and IgG using the ELISA method also yielded negative results. Two additional mothers had symptomatic infections, documented by positive RT-PCR, but did not have positive antibody detection 30 and 32 days from symptoms and positive PCR respectively. These findings raise questions regarding the immunogenicity of this virus and/or the reliability of the assays in neonates, as well as in pregnant women since, like the first baby, these mothers did not present any detectable antibody reaction. In a study of 173 patients with positive SARS-CoV-2 RT-PCR results, Zhao et al. (18) reported seroconversion rates of 82.7% for IgM and 64.7% for IgG. Likewise, Jin et al. (19) examined a population of 43 patients with confirmed infections and 33 patients with suspected infections, and reported sensitivity rates of 48.1% for IgM and 88.9% for IgG. These studies did not include pregnant women or newborn infants, who may have reduced immunologic reactions to the virus. In addition, antibody kinetics during COVID-19 is still matter of debate (12) and assays performance remains questionable (18, 19). Guo used an ELISA-based assay to analyze 208 plasma samples collected from 82 confirmed and 58 probable cases, and found that the median time of IgM detection was 5 days (interquartile range: 3–6 days), with no more detection 21 days after the onset of symptoms (12).

Regarding cellular immunity, Qin et al. (20) demonstrated a possible dysregulation of immune response. Of the 452 consecutive COVID-19-infected patients at Tongji hospital over 1 month, 286 were diagnosed as having severe infection. Severe cases tended to exhibit lower lymphocytes counts, higher leukocytes count, and lower percentages of monocytes, eosinophils, and basophils. At birth, our presently reported infants did not exhibit altered blood count cells, and did not show severe symptomatology. The observed absence of humoral immunologic responses to viral infection in these cases raise questions regarding the reliability of the tests, and the possibility of variable immunogenicity of this new virus (12, 18, 19). Moreover, it seems likely that pauci-symptomatic COVID-19-infected individuals display lower viral loads than severe cases, and may thus generate lower levels and different patterns of antibodies (21).

In conclusion, this case series raise caution regarding the risk of contamination by asymptomatic newborns that may excrete the virus in their respiratory secretion and stools. Because of the lack of reliability of antibody detection in COVID-19 infected pregnant women, all infants born from mothers with a remote history of COVID-19 should be tested within the 1st days of life regardless of their mother immunologic status.

## Data Availability Statement

The raw data supporting the conclusions of this article will be made available by the authors, without undue reservation.

## Ethics Statement

Ethical review and approval was not required for the study on human participants in accordance with the local legislation and institutional requirements. Written informed consent to participate in this study was provided by the participants' legal guardian/next of kin. Written informed consent was obtained from the minor(s)' legal guardian/next of kin for the publication of any potentially identifiable images or data included in this article.

## Author Contributions

J-MH designed the study report, performed literature search, collected the data, made data interpretation, and wrote the first draft of the manuscript. J-MJ took care of the infants and equally collected the data of the neonates. CH equally performed viral RT-PCR analysis and antibody detection. AW took care of the infants and equally collected the data of the neonates. HJ equally performed viral RT-PCR analysis and antibody detection, performed literature search, made immunology data interpretation. PF coordinated laboratory analysis. OM collected the data of the mothers. All authors equally revised and approved the submitted manuscript.

## Conflict of Interest

The authors declare that the research was conducted in the absence of any commercial or financial relationships that could be construed as a potential conflict of interest.
